# Supplementary material for: Widespread PREMA in the upper mantle indicated by low-degree basaltic melts
Source: Nat Commun. 2023 Dec 9;14:8150. doi: 10.1038/s41467-023-43845-4 (PMC10710414; doi:10.1038/s41467-023-43845-4)
Supplement: Supplementary file 1 — Supplementary Information [file 41467_2023_43845_MOESM1_ESM.pdf]

Supplementary Figures for
Widespread PREMA in the upper mantle indicated by low-degree basaltic melts

Ronghua Cai [1], Jingao Liu [1*], D. Graham Pearson [2], Andrea Giuliani [3], Peter E. van Keken [4], Senan Oesch [3]

1. State Key Laboratory of Geological Processes and Mineral Resources, China University of Geosciences, Beijing 100083, China
2. Department of Earth and Atmospheric Sciences, University of Alberta, Edmonton, Alberta T6G 2E3, Canada
3. Institute of Geochemistry and Petrology, Department of Earth Sciences, ETH Zurich, 8092 Zurich, Switzerland
4. Earth and Planets Laboratory, Carnegie Institution for Science, Washington, DC 20015, USA

Content: Fig. S1 to S10

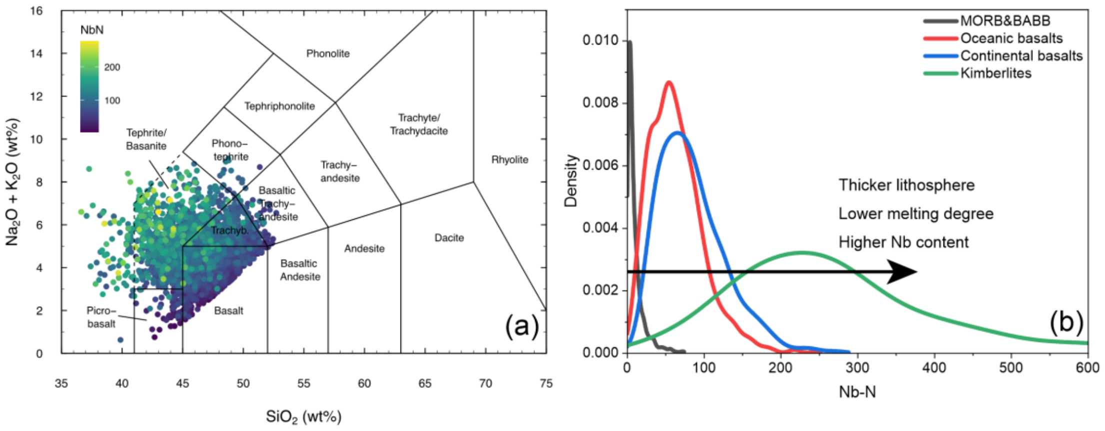

Fig. S1 (a) Total alkali vs. silica diagram of Cenozoic sodic basalts compiled in this study. (b) Density curves of Nb-N of MORB&BABB (back-arc basin basalts), oceanic and continental basalts and kimberlites.

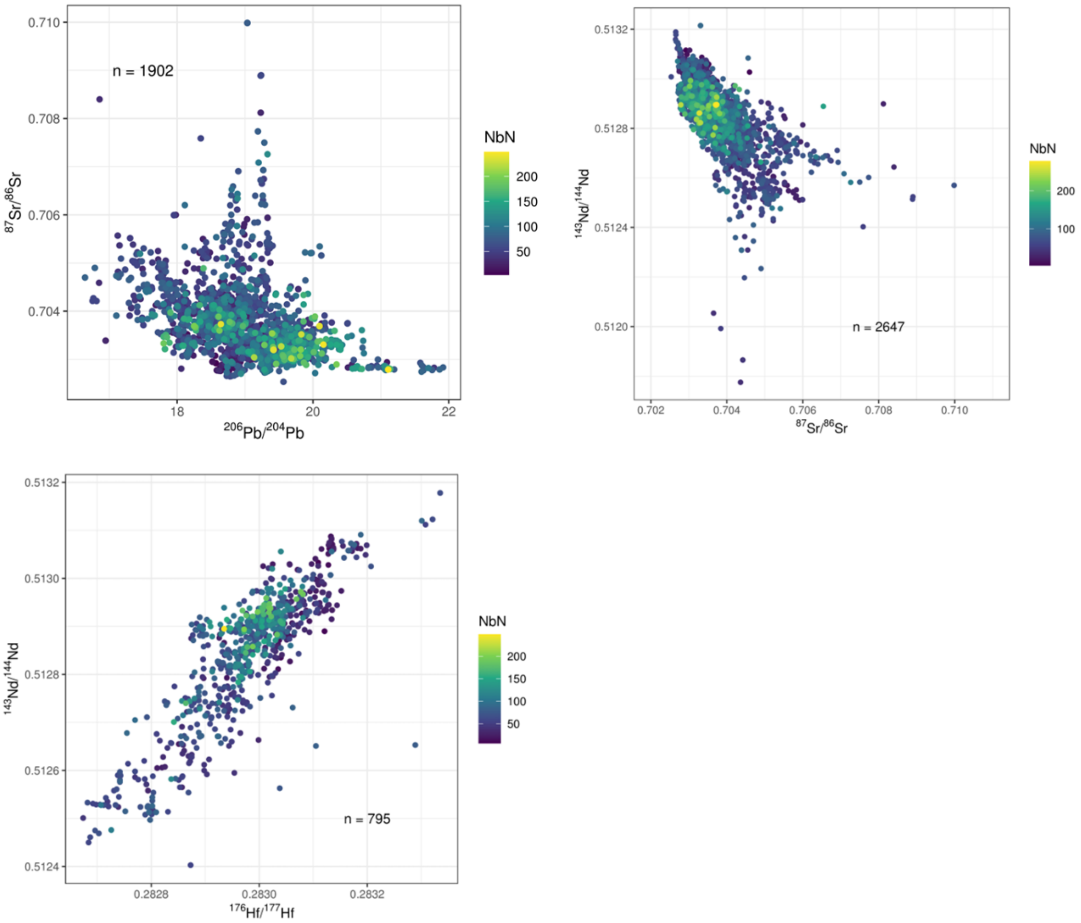

Fig. S2 Sr-Nd-Pb-Hf isotope of global Cenozoic sodic basalts.

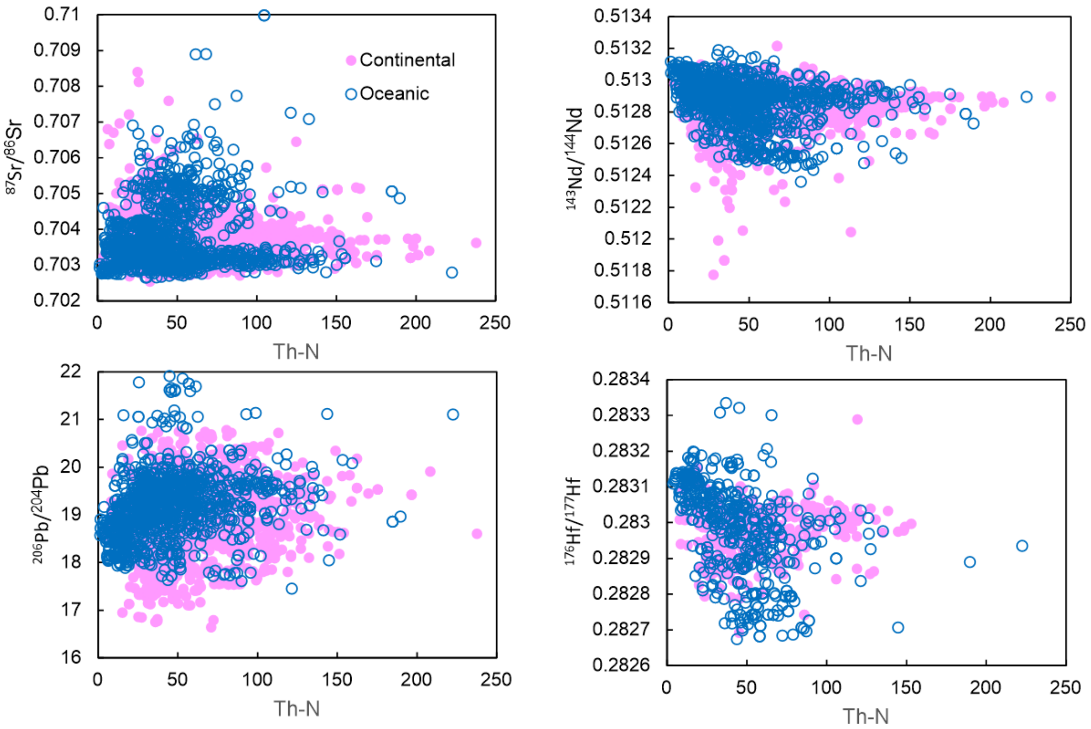

Fig. S3 Sr-Nd-Pb-Hf isotope vs Th-N of global Cenozoic sodic basalts. Th-N represents Th concentrations normalised to the primitive mantle value

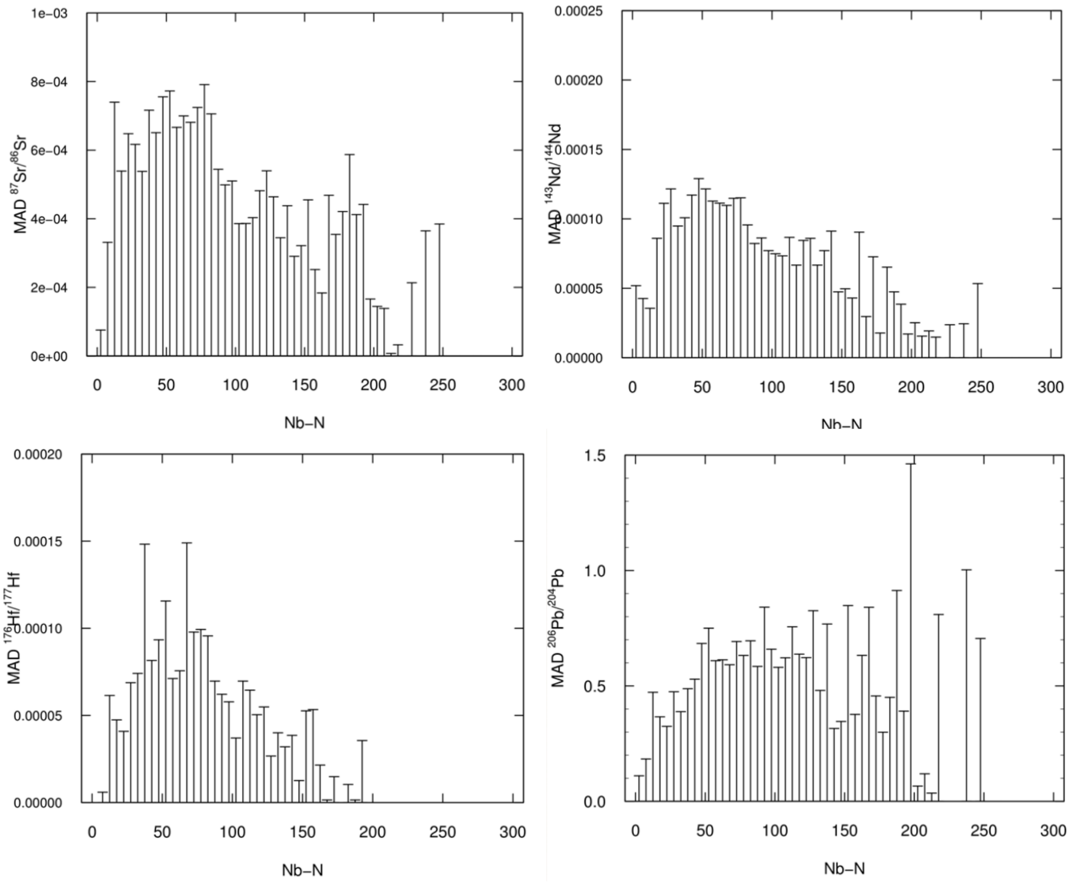

Fig. S4 Median absolute deviations (MADs) of Sr-Nd-Hf isotope ratios of global Cenozoic sodic basalts.

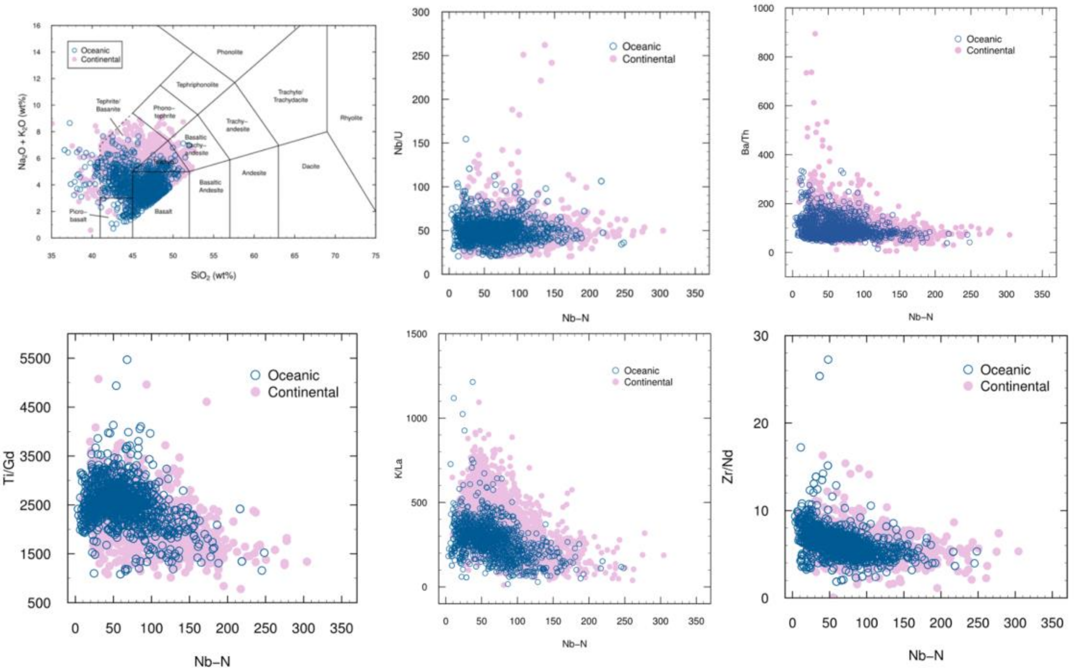

Fig. S5 Total alkali vs. silica diagram and element ratio vs. Nb-N diagrams of Cenozoic sodic basalts compiled in this study.

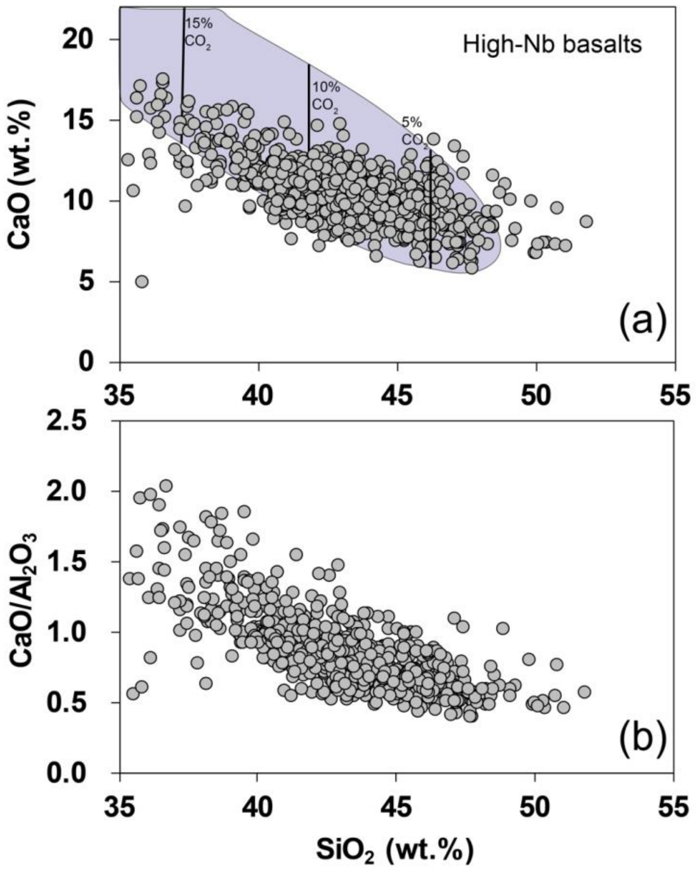

Fig. S6 SiO_2 contents versus CaO contents (a) and CaO/Al_2O_3 ratios (b) diagrams of high-Nb basalts. The shaded region contains experimental melts from a carbonated source, and the black lines indicate the stated percentage of CO_2 in the primary melts (modified from ref. 1).

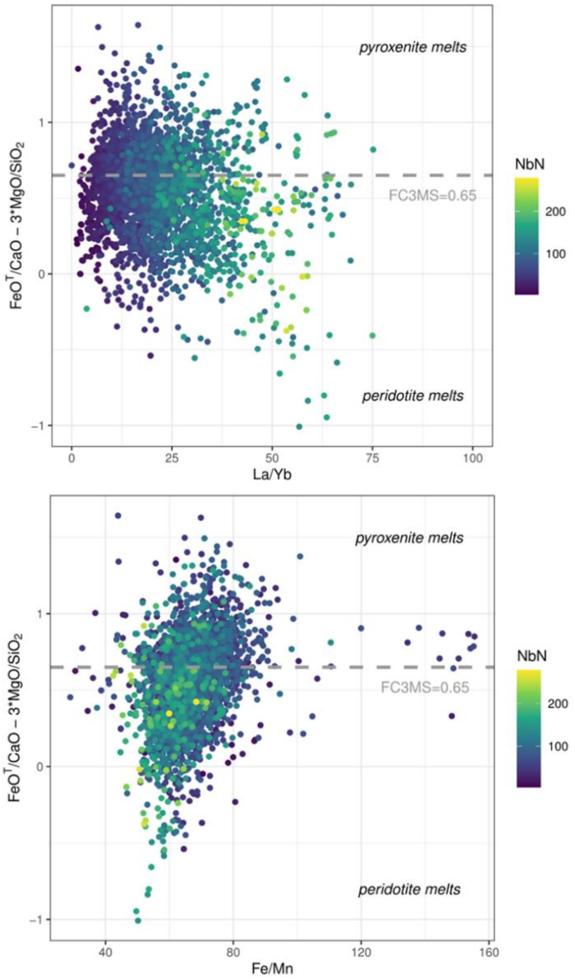

Fig. S7 FC3MS vs. Fe/Mn and La/Yb diagrams. To assess the contribution of pyroxenite in the source, we used the FC3MS parameter (FeO/CaO-3*MgO/SiO_2, all in wt%) suggested by ref. 2.

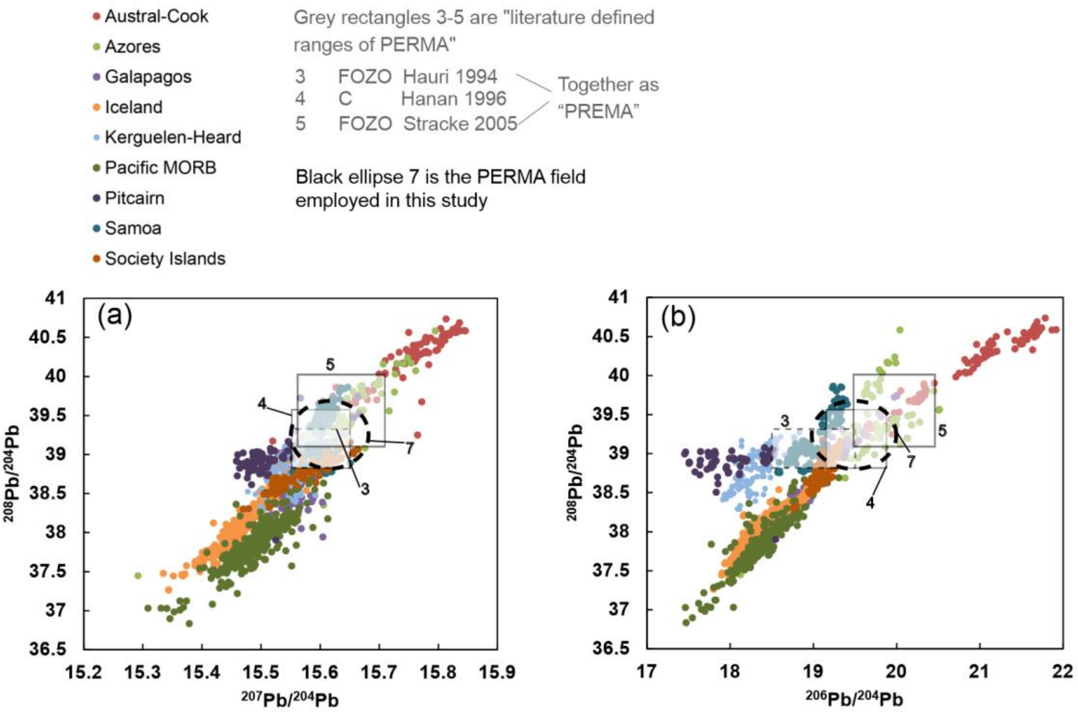

Fig. S8 Present-day Pb isotope compositions of MORBs and OIBs. (a) ^{207}Pb/^{204}Pb vs. ^{208}Pb/^{204}Pb; (b) ^{206}Pb/^{204}Pb vs. ^{208}Pb/^{204}Pb. The black rectangles represent the range of "PREMA" defined in the literature ref. 3–5 while the black ellipses indicate the "PREMA" field employed in this study. The data of MORBs and OIBs are from ref. 6. The legends are same as Fig. 1.

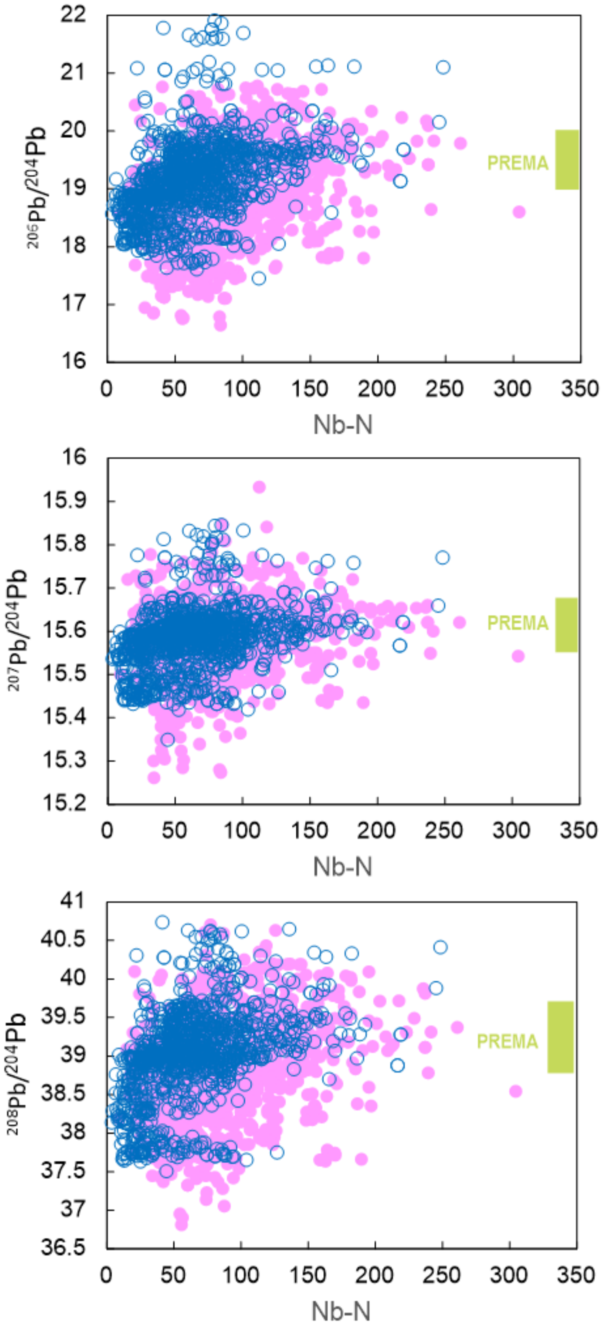

Fig. S9 Covariation of plots of Nb-N vs Pb isotopes for Cenozoic sodic basalts from continental (pink) and oceanic (blue) domains. The PREMA bar is taken from the range proposed by this study (Fig. 1, S8).

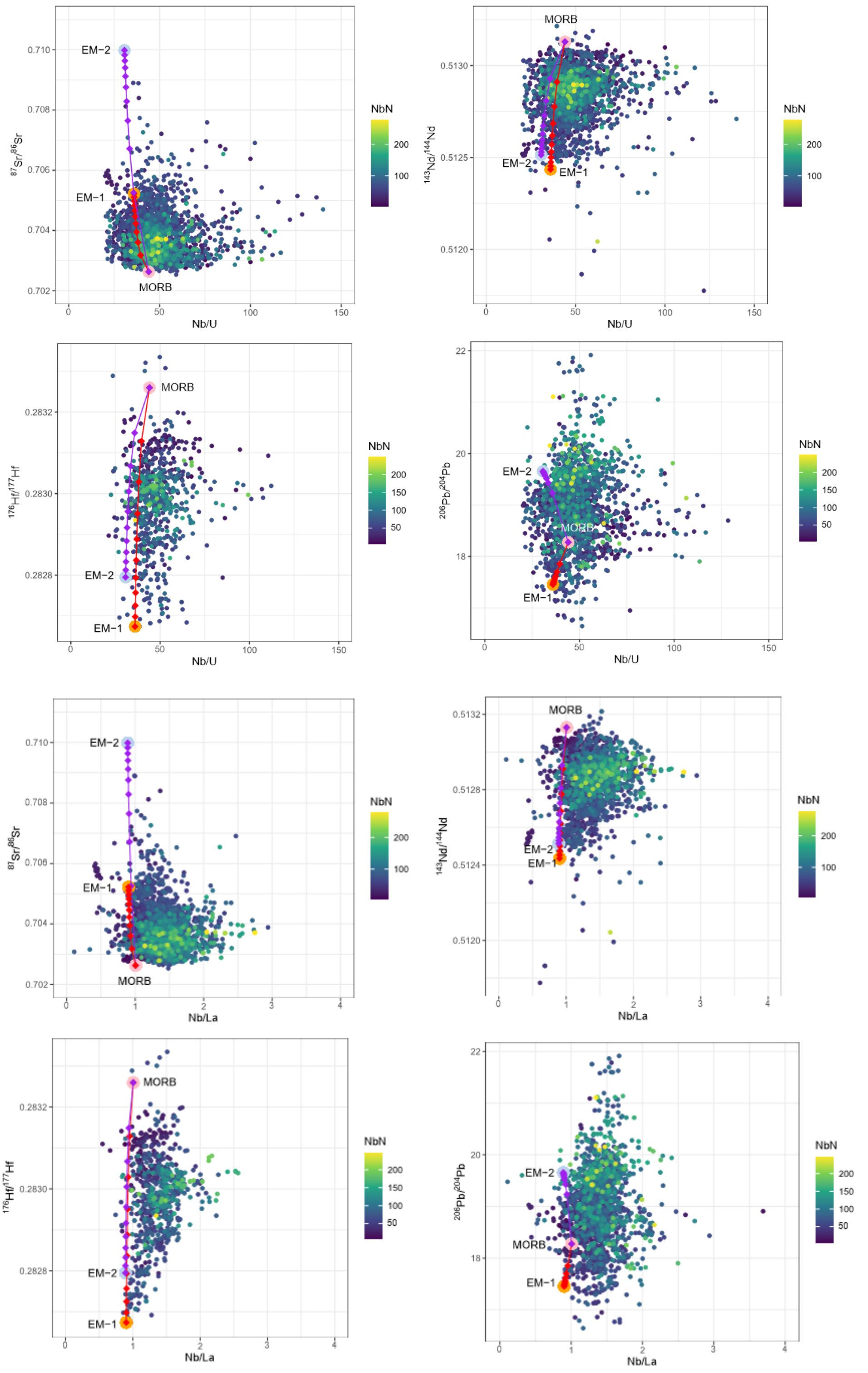
EM−2
EM−1
MORB
$^{87}Sr/^{86}Sr$
Nb/U
NbN
$^{143}Nd/^{144}Nd$
$^{176}Hf/^{177}Hf$
$^{206}Pb/^{204}Pb$
Nb/La
0.710
0.708
0.706
0.704
0.702
0.5130
0.5125
0.5120
0.5132
0.5128
0.5124
0.2832
0.2830
0.2828
22
20
18
0
50
100
150
200
1
2
3
4

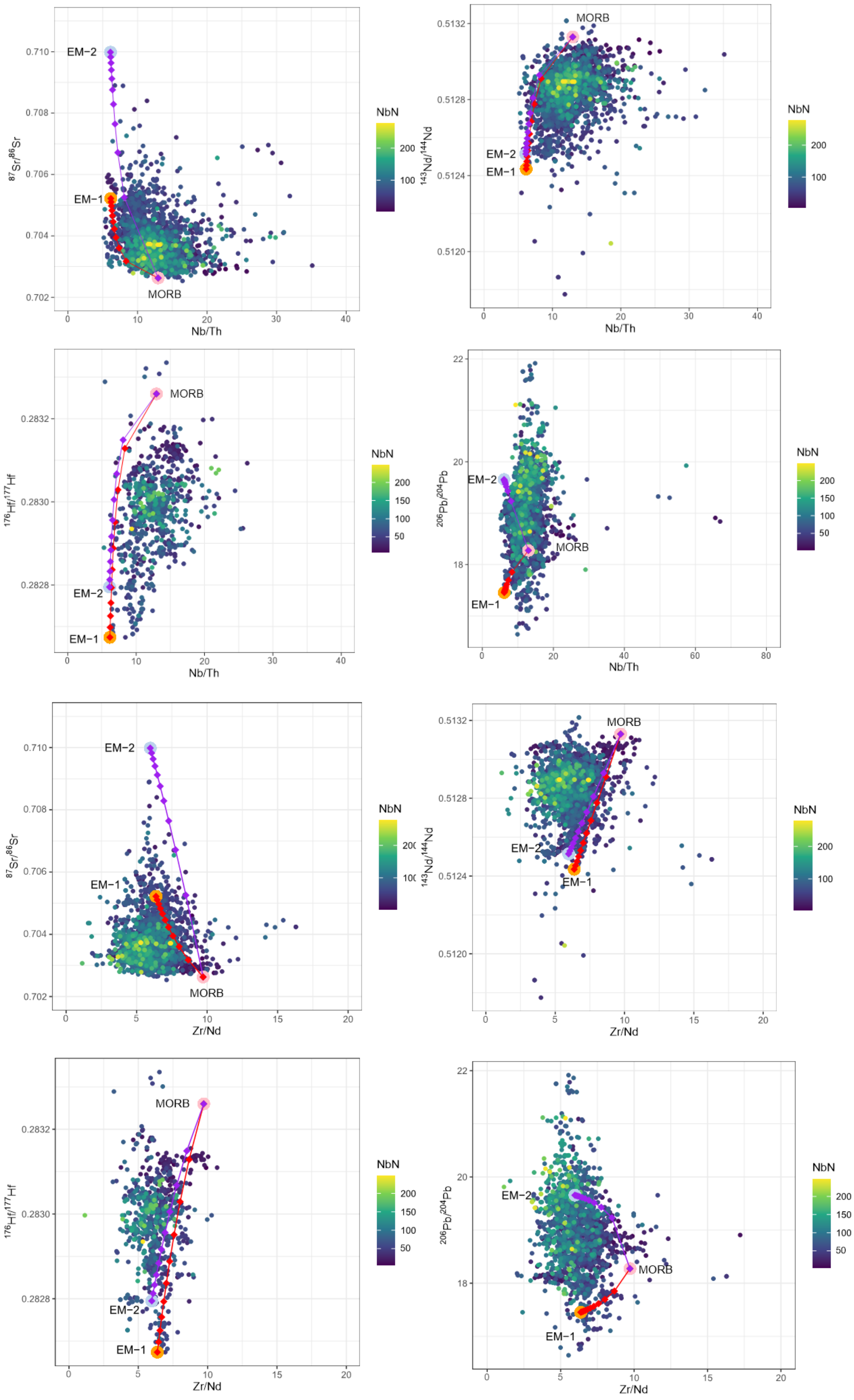

EM−2
EM−1
MORB
NbN
200
100
$^{87}Sr/^{86}Sr$
0.710
0.708
0.706
0.704
0.702
0
10
20
30
40
Nb/Th
$^{143}Nd/^{144}Nd$
0.5132
0.5128
0.5124
0.5120
$^{176}Hf/^{177}Hf$
0.2832
0.2830
0.2828
150
50
$^{206}Pb/^{204}Pb$
22
20
18
60
80
Zr/Nd
5
15

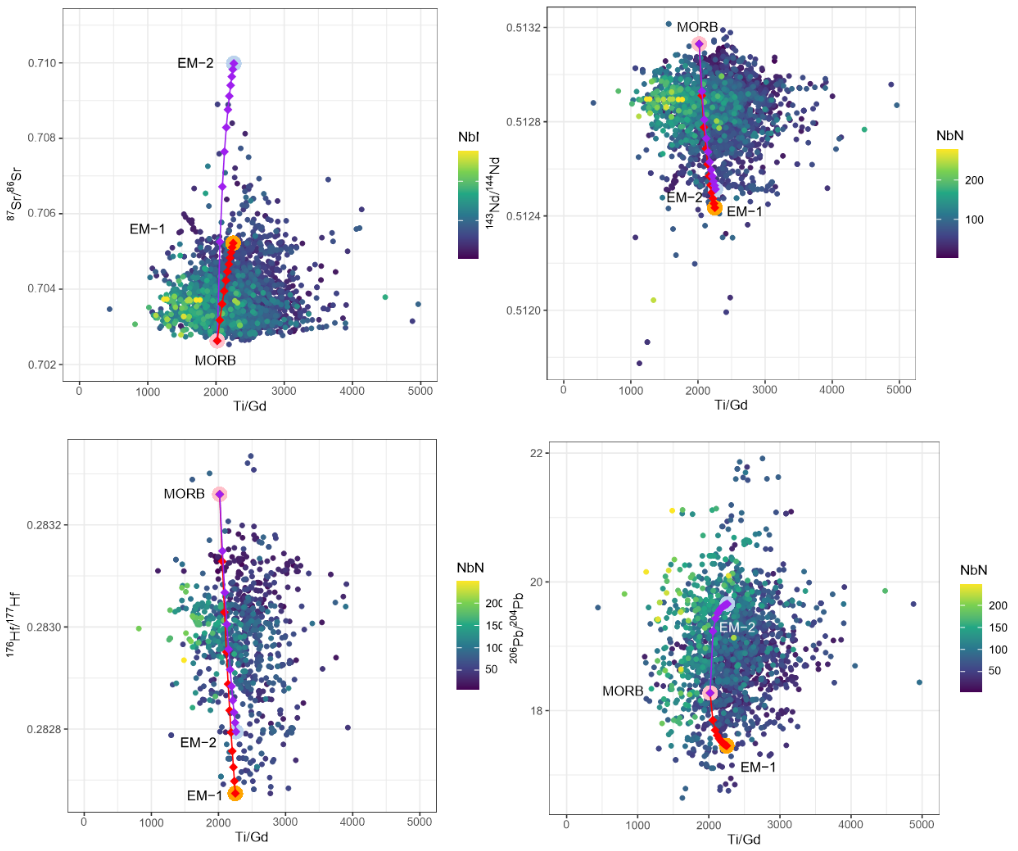

Fig. S10 Covariation diagrams of Nb/U, Nb/La, Nb/Th, Zr/Nd, Ti/Gd vs radiogenic isotope ratios for global Cenozoic sodic basalts colour-coded based on Nb-N. The details of the mixing curves are the same as in Fig. 6.

Supplementary References

1. Mazza, S. E. et al. Sampling the volatile-rich transition zone beneath Bermuda. Nature 569, 398–403 (2019).
2. Yang, ZF., Zhou, JH. Can we identify source lithology of basalt?. *Sci Rep* **3**, 1856 (2013). https://doi.org/10.1038/srep01856
3. Hanan, B. B. & Graham, D. W. Lead and helium isotope evidence from oceanic basalts for a common deep source of mantle plumes. Science 272, 991–995 (1996).
4. Hauri, E. H., Whitehead, J. A. & Hart, S. R. Fluid dynamic and geochemical aspects of entrainment in mantle plumes. J. Geophys. Res. 99, (1994).
5. Stracke, A., Hofmann, A. W. & Hart, S. R. FOZO, HIMU, and the rest of the mantle zoo. *Geochem. Geophys. Geosyst.* **6**, (2005).
6. Stracke, A. Earth's heterogeneous mantle: A product of convection-driven interaction between crust and mantle. Chem. Geol. 330–331, 274–299 (2012).
