# Supplementary material for: Widespread PREMA in the upper mantle indicated by low-degree basaltic melts
Source: Nat Commun. 2023 Dec 9;14:8150. doi: 10.1038/s41467-023-43845-4 (PMC10710414; doi:10.1038/s41467-023-43845-4)
Supplement: Supplementary file 3 — Description of Additional Supplementary Files [file 41467_2023_43845_MOESM3_ESM.pdf]

<u>Description of Additional Supplementary Files</u>

Supplementary Data 1: Global Cenozoic basalt data compiled in this study

Supplementary Movie 1: Global-scale mantle-convection model
